# An Integrative Omics Strategy to Assess the Germ Cell Secretome and to Decipher Sertoli-Germ Cell Crosstalk in the Mammalian Testis

**DOI:** 10.1371/journal.pone.0104418

**Published:** 2014-08-11

**Authors:** Frédéric Chalmel, Emmanuelle Com, Régis Lavigne, Nolwen Hernio, Ana-Paula Teixeira-Gomes, Jean-Louis Dacheux, Charles Pineau

**Affiliations:** 1 IRSET, Inserm U1085, Campus de Beaulieu, Rennes, France; 2 Proteomics Core Facility Biogenouest, Inserm U1085 IRSET, Campus de Beaulieu, Rennes, France; 3 INRA UMR 1282, Infectiologie et Santé Publique, Nouzilly, France; 4 INRA Plate-forme d'Analyse Intégrative des Biomolécules (PAIB^2^), Nouzilly, France; 5 UMR 85 INRA-CNRS, Nouzilly, France; University of Nevada School of Medicine, United States of America

## Abstract

Mammalian spermatogenesis, which takes place in complex testicular structures called seminiferous tubules, is a highly specialized process controlled by the integration of juxtacrine, paracrine and endocrine information. Within the seminiferous tubules, the germ cells and Sertoli cells are surrounded by testicular fluid (TF), which probably contains most of the secreted proteins involved in crosstalk between these cells. It has already been established that germ cells can modulate somatic Sertoli cell function through the secretion of diffusible factors. We studied the germ cell secretome, which was previously considered inaccessible, by analyzing the TF collected by microsurgery in an “integrative omics” strategy combining proteomics, transcriptomics, genomics and interactomics data. This approach identified a set of proteins preferentially secreted by Sertoli cells or germ cells. An interaction network analysis revealed complex, interlaced cell-cell dialog between the secretome and membranome of seminiferous cells, mediated via the TF. We then focused on germ cell-secreted candidate proteins, and we identified several potential interacting partners located on the surface of Sertoli cells. Two interactions, APOH/CDC42 and APP/NGFR, were validated *in situ*, in a proximity ligation assay (PLA). Our results provide new insight into the crosstalk between germ cells and Sertoli cells occurring during spermatogenesis. Our findings also demonstrate that this “integrative omics” strategy is powerful enough for data mining and highlighting meaningful cell-cell communication events between different types of cells in a complex tissue, via a biological fluid. This integrative strategy could be applied more widely, to gain access to secretomes that have proved difficult to study whilst avoiding the limitations of *in vitro* culture.

## Background

Mammalian spermatogenesis, which takes place within the seminiferous tubules, is a multistep process conserved between species and playing a crucial role in the transmission of genetic heritage. Spermatogenesis can be split into three phases on the basis of anatomical and biochemical features: a proliferative or mitotic phase, in which the primitive germ cells – spermatogonia – renew themselves and undergo a series of mitotic divisions; the meiotic phase, in which the diploid spermatocytes undergo two consecutive divisions to produce haploid spermatids; and spermiogenesis, in which the spermatids develop into spermatozoa [1].

This unique process is controlled by juxtacrine, paracrine and endocrine factor signals, and is conditioned by the successive activation and/or repression of thousands of genes and proteins, including many testis-specific isoforms [for reviews, see [2]–[7]. All these features make the testis one of the most complex organs in the body [3] and this complex physiological structure creates particular difficulties for studies of testis organization, function and regulation. Studies of the interactions between Sertoli and germ cells are challenging, due to the anatomical complexity and probable interdependence of these cells.

Sertoli and germ cells probably communicate through a unique set of structural devices and functional interactions [2], [8]. Sertoli cells were first described in 1865 [9] and are known to have nursing properties. They supply the germ cells, at all stages of development, with the factors they need for their division, differentiation and metabolism. They are also thought to help germ cells to synchronize their development and to help maintain the wave of spermatogenesis [for a review, see [3]]. Conversely, germ cells have been shown to regulate Sertoli cell function, in both *in vivo* and *in vitro* studies. Since the late 1980s, the influence of germ cells has been known to be exerted through cell-cell contacts, via cytoplasmic structures allowing the transfer of germ cell materials [for a review see [3]] and the secretion of diffusible, proteinaceous factors [10]–[13]. However, differentiated germ cells have proved impossible to maintain *in vitro*, making it very difficult to study their secretome. For this reason, the role of these cells in spermatogenesis control has been largely neglected since it first emerged in the early 1990s [for a review see [3]].

The absence of such an “experimental testing ground” makes genome-wide “omics” approaches even more important. Significant progress has been made in the large-scale analysis of high-throughput data greatly increasing our knowledge of spermatogenesis, by making it possible to identify hundreds of genes displaying spatial and temporal regulation during the testicular ontogenesis essential for the differentiation of male gametes [for reviews, see [14]–[16]]. Our understanding of normal and pathological spermatogenesis has been greatly increased by the use of transcriptomics (microarrays and, more recently, RNA-sequencing technologies) and proteomics (differential and shotgun technologies) [for review see, [17]–[20]]. Nevertheless, with few exceptions [18], [20]–[23], very little effort has been made to combine the resulting data in a cross-/multispecies integrative “omics” approach, to address specific biological questions relating to spermatogenesis.

The two compartments of the testis are immersed in different fluids. Interstitial tissue is immersed in the interstitial fluid that is derived from blood as a capillary filtrate. Seminiferous tubules are immersed in, the seminiferous or testicular fluid (TF). Sertoli cells contribute to the production of the TF that surrounds the seminiferous cells and contains hundreds of peptides, proteins and steroid hormones [24], [25], which may be involved in crosstalk between germ cells and Sertoli cells. We developed an innovative strategy to enable us to study and decipher the germ cell secretome, with the aim of unraveling the molecular mechanisms underlying the crosstalk between Sertoli and germ cells. The TF proteomes of two species were analyzed by shotgun mass spectrometry and the results obtained were combined with those of transcriptome and theoretical secretome analyses on testicular seminiferous cells. This approach identified a set of dozens of genes encoding proteins present in the TF and potentially secreted by either Sertoli or germ cells. The integration of interactomics data then made it possible to detect potential interacting partners located on the surface of either germ cells or Sertoli cells. For the validation of our screening approach and candidate selection, we focused on two protein-protein interactions, which were confirmed *in situ* on rat testis sections, in proximity ligation assays (PLA).

## Results

### Experimental design and workflow

**Figure 1:**
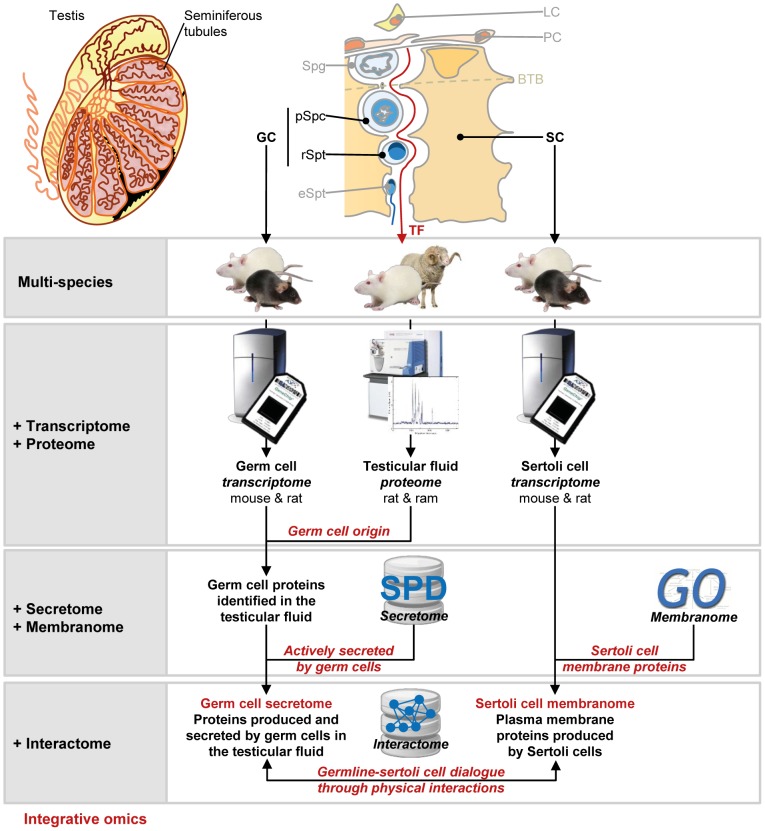
Experimental design and integrative omics workflow. A schematic diagram of the strategy used to access germ cell and Sertoli cell secretomes and to highlight potential protein-protein interactions. BTB: blood testis barrier; GC: germ cells; LC: Leydig cells; PC: peritubular cells; SC: Sertoli cells; Spg: spermatogonia; pSpc: pachytene spermatocytes; rSpt: round spermatids; eSPT: elongated spermatids.

The primary objective of this study was to decipher the testicular germ cell secretome, which had previously been inaccessible, by analyzing the TF. The secondary objective was to highlight key proteins potentially involved in dialog between Sertoli and germ cells, focusing particularly on the proteins secreted by germ cells and involved in the regulation of Sertoli cell functions. We addressed these issues, by establishing a cross-species “integrative omics” workflow combining several types of large-scale data, as presented in Fig. 1. We first determined the core mammalian TF proteome, which we assumed would contain most of the diffusible factors involved in cell-cell crosstalk. We collected TF from male rats and rams. The TF was then fractionated and analyzed by shotgun proteomics methods, to identify as many of the proteins present in these complex biological fluids as possible. We used a gene expression dataset including the Sertoli and germ cell transcriptomes [26], to identify the candidate proteins unambiguously originating from particular seminiferous cell populations. We then focused on those genes preferentially expressed in one testicular cell type for which the corresponding gene product had been identified in the TF and that were known to encode actively secreted proteins; these genes were identified with the Secreted Protein Database [SPD; [27]]. In parallel, by combining the same seminiferous cell transcriptome dataset and the set of loci encoding plasma membrane or cell surface proteins, we assembled the individual testicular cell membranomes. We finally investigated whether physical protein-protein interactions between members of the Sertoli or germ cell secretome and members of the germ cell or Sertoli cell membranome had already been reported in other biological systems, using interactomic data from public repositories [see Materials and Methods; [28]–[31]].

### Defining the mammalian TF proteome

We analyzed, compared and combined the sets of proteins identified in the TF of two mammalian species, *Rattus norvegicus* (rat) and *Ovis aries* (sheep), to build a reference map of the TF proteome in mammals. The rat is an established model organism for reproductive biology and toxicology, whereas the ram is a model of choice for studies on the hormonal control of reproduction [32]–[36]. The ram, with its large, accessible testes, was considered an ideal model for this project, as up to 20 to 25 ml of TF can be collected per testis in a given experiment from rams, whereas only 2 to 6 µl of TF per testis can be collected from rats.

**Figure 2:**
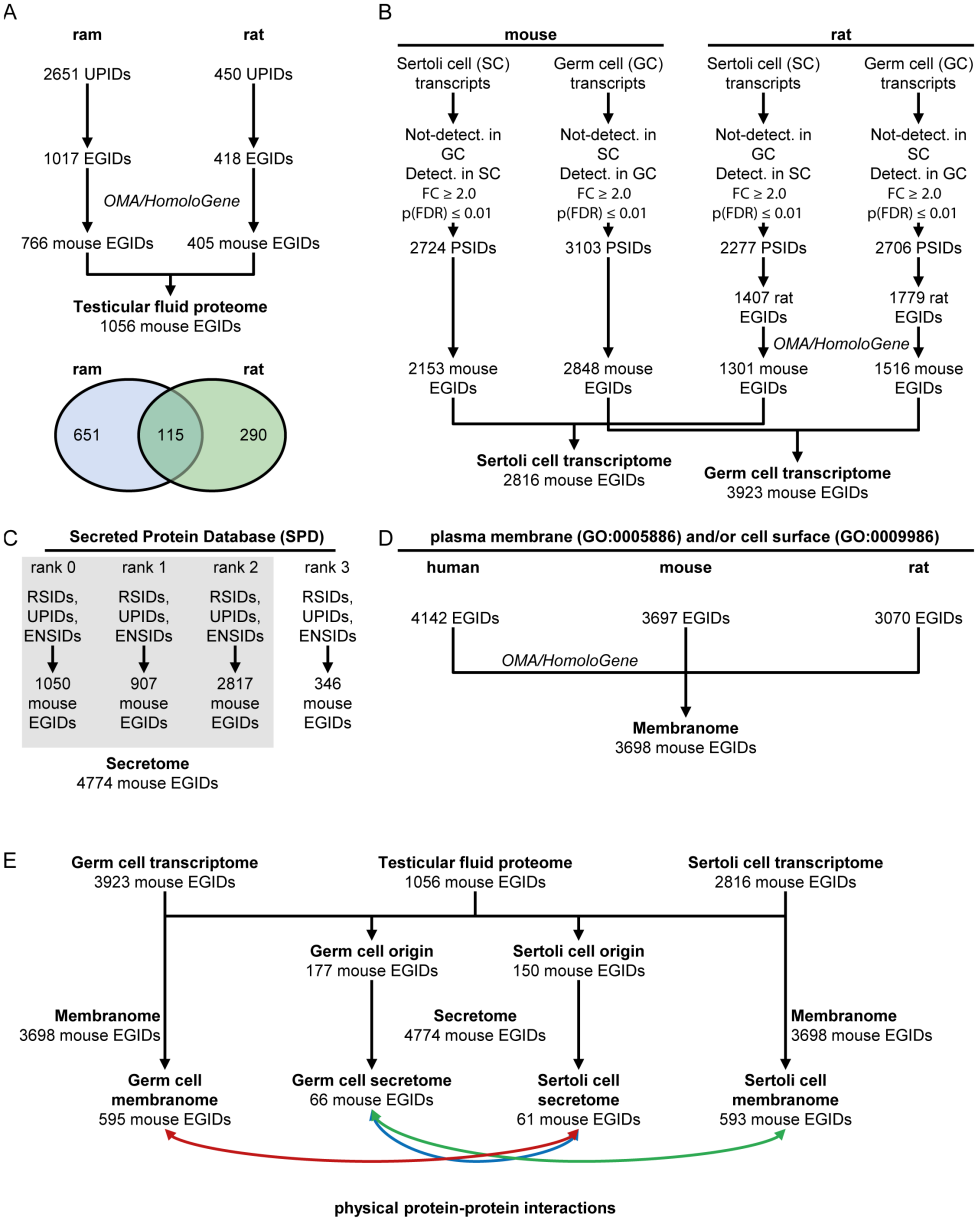
Details of datasets and the methods used to reconstruct dialog between Sertoli and germ cells. (A) Conversion of the rat and ram UniProt identifiers (UPIDs) into mouse Entrez Gene identifiers (EGIDs). (B) Definition of the germ cell and Sertoli cell transcriptomes (PSIDs: Probeset identifiers) (C) Selection of loci (mouse Entrez gene IDs) encoding secreted or potentially secreted proteins (RSIDs: RefSeq identifiers UPIDs: UniProt identifiers; ENSIDs: Ensembl identifiers) (D) Selection of genes encoding proteins associated with a “plasma membrane” and/or “cell surface” location. (E) Selection of proteins secreted by one type of cell (germ or Sertoli cell) and interacting with membrane proteins of the other type of cell from BioGRID, HPRD, IntAct, MINT and NCBI databases.

We maximized the chances of picking up low-copy number proteins, by fractionating rat and ram TF before shotgun mass spectrometry analysis, by SDS-PAGE, after which the gel lanes were divided into 20 equal-sized pieces. The gel fragments were subjected to trypsin digestion and the resulting peptide digests were analyzed by nano-LC-MS/MS, generating 20 subproteomes. Overall, we identified 2,651 proteins in ram TF and 450 proteins in rat TF (UniProt identifiers (IDs)) [Fig. 2, panel A; PRIDE Proteomics Identifications database (http://www.ebi.ac.uk/pride); accession number: 31052-31111].

For direct comparisons of the two lists of gene products, the resulting UniProt IDs were sequentially matched with the corresponding gene entries (NCBI Entrez gene IDs), orthologous entries (HomoloGene IDs) and *Mus musculus* gene IDs. This strategy made it possible to decrease the redundancy of the identified proteins in a significant, unambiguous manner. We found that the 2,651 ram TF proteins and 450 rat TF proteins identified corresponded to 766 and 405 mouse genes, respectively (Entrez gene IDs). We next merged the two mouse gene lists and identified a total of 1,056 proteins (mouse Entrez gene IDs) present in TF from rat or ram. This dataset constitutes the first reference proteome for the mammalian TF (Fig. 2, panel A and table S1).

### One sixth of the proteins identified in TF were preferentially produced by meiotic and post-meiotic germ cells

The TF is generated principally by Sertoli cells [37]. Sertoli cells clearly secrete active compounds into this fluid, but the release of soluble factors into the TF by germ cells has never been directly studied and documented. We investigated the contributions of the two cell types to TF content, by comparing the mammalian TF proteome characterized here with a previously published transcriptome [26] obtained with highly enriched populations of Sertoli cells, pachytene spermatocytes and round spermatids, from both mice and rats. We sought to integrate this expression dataset into our data, to distinguish between proteins originating preferentially from either the Sertoli cells or from the meiotic/post-meiotic germ cells. We first identified mouse and rat genes preferentially expressed in Sertoli cells rather than meiotic/post-meiotic germ cells, and in meiotic/post-meiotic germ cells rather than Sertoli cells (Fig. 2, panel B, see Materials and Methods). The rat gene IDs obtained for the rat expression dataset were converted into the corresponding mouse gene IDs. This statistical filtering process led to the definition of a germ-cell reference transcriptome composed of 3,923 mouse genes displaying significant differential expression between meiotic/post-meiotic germ cells and Sertoli cells. We also defined the Sertoli cell reference transcriptome, which consisted of 2,816 loci more strongly expressed in Sertoli cells than in germ cells (Fig. 2, panel B). We next focused on a subset of 177 loci preferentially expressed in germ cells (the germ-cell reference transcriptome) and encoding proteins present in the TF (the mammalian TF reference proteome) (Fig. 2, panel E). We then applied the same strategy to identify a subset of 150 genes preferentially expressed by Sertoli cells (i.e. present in both the Sertoli reference transcriptome and the TF reference proteome) (Fig. 2, panel E). About one sixth (177/1,056) of the 1,056 genes encoding proteins identified in the TF were preferentially expressed in meiotic/post-meiotic germ cells, and about one seventh (150/1,056) were preferentially expressed in Sertoli cells (table S1).

### The set of proteins originating from germ cells and identified in TF displays significant enrichment in secreted factors

One of the key issues in this integrative genomics approach was determining the extent to which combining the TF reference proteome and the germ-cell (or Sertoli cell) reference transcriptome could help to identify the diffusible factors produced by germ cells (or Sertoli cells) and secreted into the TF. We therefore incorporated into the analysis a set of secreted factors from the SPD, in which the proteins are ranked according to a prediction confidence score of 0 to 3 [27]. We defined the reference secretome as mouse genes encoding proteins of ranks 0–2. This reference secretome consisted of 4,774 genes encoding known or predicted diffusible factors (Fig. 2, panel C; see Materials and Methods).

As expected, a significant proportion of the 747 loci encoding secreted proteins preferentially produced by germ cells was retrieved in the TF (66/177, *p*<4.6×10^−9^) (Fig. 2, panel E). Similarly, the list of genes expressed by Sertoli cells and encoding proteins identified in the TF was significantly enriched in genes encoding diffusible factors (61/150, *p*<3.1×10^−6^) (Fig. 2, panel E).

### Prediction of the core molecular interactome, providing information about the cell-cell dialog occurring within the seminiferous tubules

We investigated potential physical protein-protein interactions between the secreted factors of germ cells and the membrane proteins of Sertoli cells, and vice versa. We first defined the germline and Sertoli cell membranomes, by selecting genes encoding cell surface proteins (Fig. 2, panel D) from the list of genes present in the reference transcriptome for the corresponding cell type (Fig. 2, panel B). We found that about 15% (595/3,923) and 21% (593/2,816) of the loci preferentially expressed in germ cells and Sertoli cells, respectively, were associated with gene products located on the plasma membrane (Fig. 2, panel E).

**Figure 3:**
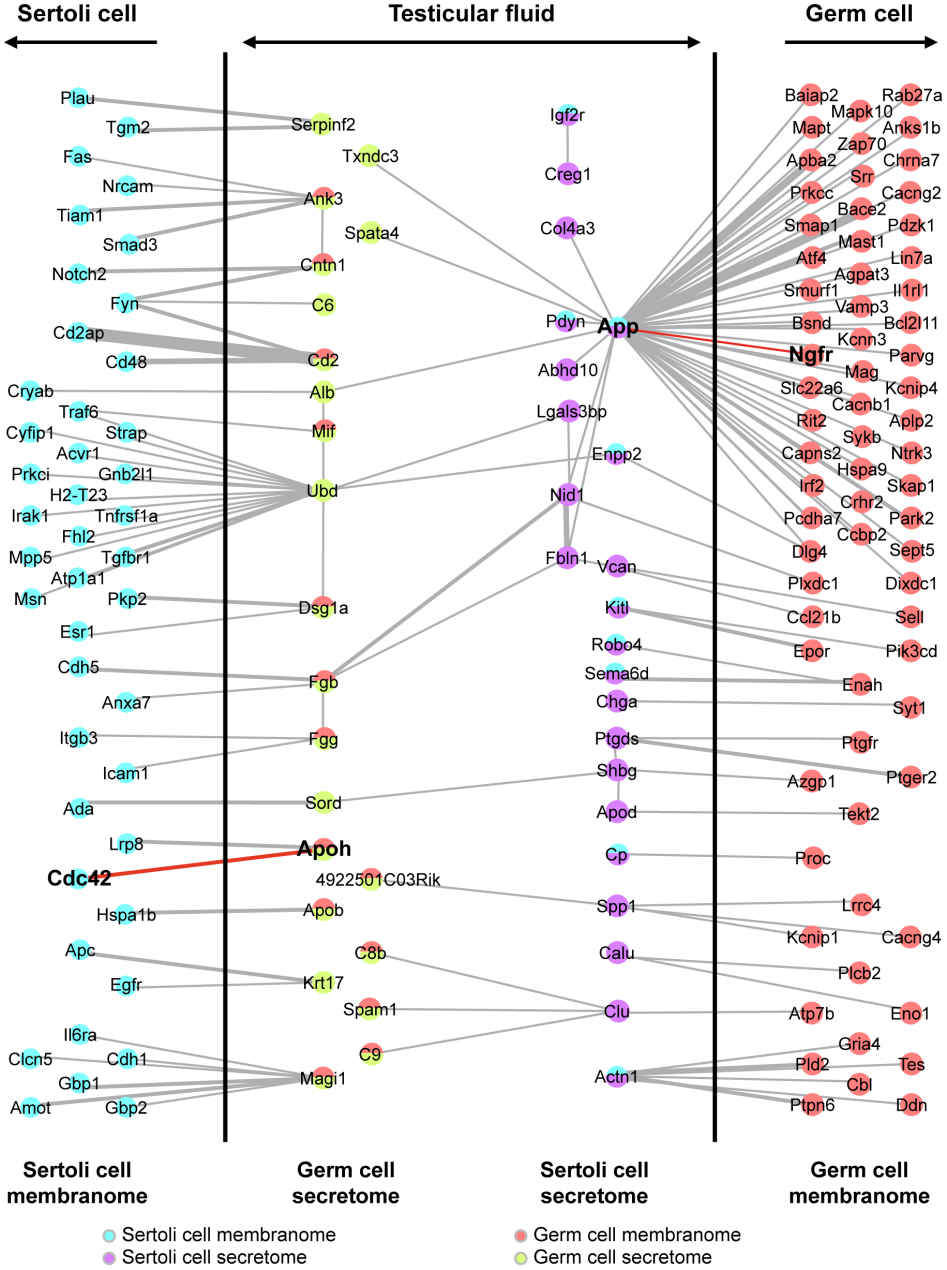
Integration of “omics” data to establish a network of molecular interactions between germ cells and Sertoli cells mediated by the TF. This integrated network focuses on proteins produced and secreted by germ cells (GCs) or Sertoli cells (SCs) and interacting with membrane proteins of the other type of cell. Nodes symbolizing GC-secreted and Sertoli cell-secreted factors are indicated in light green and purple, respectively, whereas GC-membrane and Sertoli cell-membrane proteins are represented by red and light blue nodes, respectively.

We investigated the extent to which factors secreted by one type of cell (meiotic/post-meiotic germ cells or Sertoli cells) into the TF interacted with cell surface proteins on the other type of cell, by combining cell-specific secretome data and cell-specific membranome data with information about protein-protein interactions. A graphical display created with AMEN [38] revealed a complex interlaced network of interactions between seminiferous cell types, mediated through the TF (Fig. 3). This large network consisted of 22 germ cell-secreted and 23 Sertoli cell-secreted factors interacting with 43 Sertoli cell and 69 germ cell membrane proteins. The physical associations highlighted by this analysis were supported by the findings of 140 published studies on various biological systems and model organisms. This network analysis identified well-known connections between germ cells and somatic cells, such as the intimate association of CLU (clusterin) with SPAM1 (sperm adhesion molecule 1) [39]. CLU is one of the major proteins secreted by Sertoli cells and it has already been associated with the surface of spermatozoa [40]. By contrast, SPAM1 is an important hyaluronidase secreted and acquired by spermatozoa, and playing a key role in fertilization [41], [42].

### Validation of the protein partners potentially involved in the dialog between Sertoli and germ cells

For the validation of our approach, we focused on proteins secreted by germ cells with potential partners expressed on the Sertoli cell membrane. We then investigated *in situ* two novel physical molecular interactions that had never before been reported in the testis and for which well characterized antibodies were commercially available: the interactions of APP (amyloid beta precursor protein) with NGFR (nerve growth factor receptor) and CDC42 (cell division cycle 42) with APOH (apolipoprotein H) [43], [44]. We used the *in situ* PLA to visualize protein-protein interactions in fixed rat testis sections.

**Figure 4:**
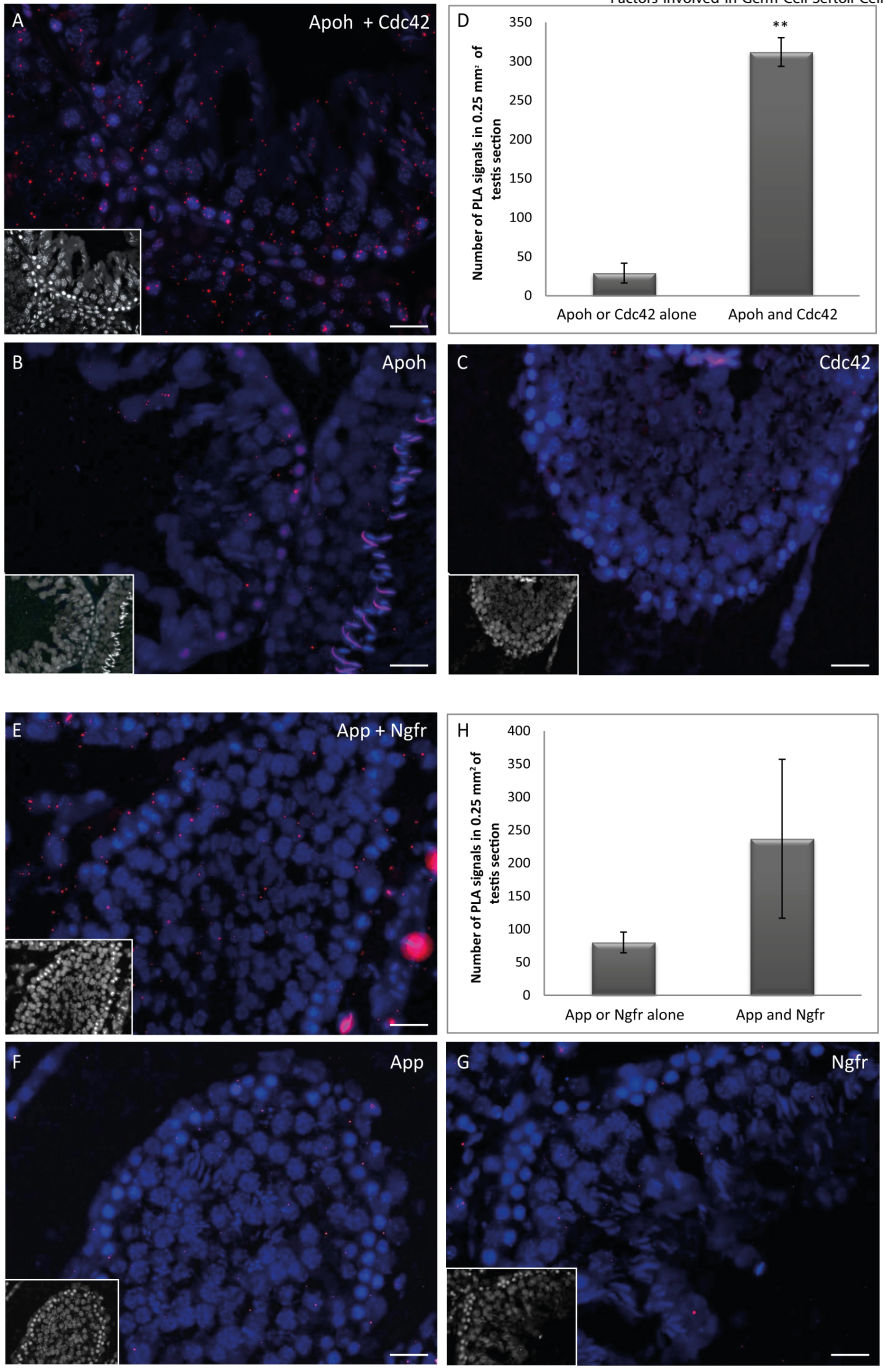
*In situ* detection of APOH/CDC42 and APP/NGFR interactions in the rat testis by Duolink PLA. (A, E) Abundant PLA signals (red dots) were detected in the seminiferous tubules of rat testis when specific primary antibodies were used, reflecting the close proximity of APOH/CDC42 or APP/NGFR close proximity. (B, C, F, G) Negative controls, with only one primary antibody for the targeted protein-protein interaction. (D, H) Quantification of PLA signals in testis sections for APOH/CDC42 (** Student's *t*-test *p* = 0.0016) or for APP/NGFR (Student's *t*-test *p* = 0.16). Scale bars = 50 µm. A nonspecific background nuclear signal was observed for a few tubule sections (see B).

The PLA can be used to visualize the molecular proximity (<4 nm) between two proteins of interest directly on tissue slices [45]. For the APOH and CDC42 proteins, abundant PLA signals (red dots) were detected on testis sections with anti-APOH and anti-CDC42 antibodies (Fig. 4A). Significantly fewer PLA signals were detected if only one primary antibody (anti-APOH or anti-CDC42) was used (Figs. 4 B and C). These observations were confirmed by the quantification of PLA signals on testis sections (Fig. 4D), which presented a mean of 312 PLA signals/0.25 mm^2^ for APOH/CDC42H interactions and 29 PLA signals/0.25 mm^2^ for APOH alone or CDC42 alone. These results were statistically significant (Student's *t*-test *p*<0.01) and strongly suggest that APOH and CDC42 interact within the rat seminiferous tubules. Intriguingly, a nuclear signal independent of the PLA signal was observed in spermatids and spermatogonia on a few tubule sections (Fig. 4B). Similar results have already been reported for fixed tissues, on which the deleterious effects of aldehydes on DNA might favor the binding of fluorescent oligonucleotides during the PLA reaction [46]. This non-specific signal, which did not appear as dots, was not taken into account when we quantified PLA signals on testis sections.

For the APP and NGFR proteins, PLA showed numerous red dots on rat testis sections with anti-APP and anti-NGFR antibodies (Fig. 4E). A smaller number of PLA signals were observed if only one of these antibodies was used (Figs. 4F and G). The counting of PLA signals confirmed this observation, with the detection of a mean of 237 PLA signals/0.25 mm^2^ in APP/NGFR conditions and of 80 PLA signals/0.25 mm^2^ for APP alone or NGFR alone (Fig. 4H). These results were not statistically significant (Student's *t*-test *p* = 0.16), but they nevertheless strongly suggest that APP and NGFR interact within the rat seminiferous tubules.

## Discussion

The primary aim of this study was to decipher the mammalian testicular germ cell secretome, which had previously not been technically possible. The maintenance of isolated germ cell populations in primary culture remains unsatisfactory. Indeed, following their isolation from rat or mouse testes, classical models in this field, a large proportion of germ cells are found to have already entered a preapoptotic process, with 40 to 60% positive for Annexin V staining (Pineau *et al*., unpublished observation). Interestingly, germ cell apoptosis is a major event within the seminiferous tubules and is undoubtedly linked to the spermatogenesis outcome. Thus, intracellular proteins in the conditioned culture medium may originate from either a physiological leakage or a degradation of isolated cells during the isolation procedures. In this context, germ cell secretomes cannot be deciphered from conditioned culture medium, because this medium contains cellular debris and a large number of intracellular proteins. Unfortunately, no immortalized germ cell line is currently available to overcome these limitations. However, a promising neonatal mouse testis culture method at the gas-liquid interface makes it possible to maintain a functional spermatogenesis *in vitro*. If needed, it could be adapted to other species (e.g., rat) with minor efforts. This innovative approach should offer great potential for studying molecular mechanisms in spermatogenesis [47]–[49]. Due to the problems described above, studies on the regulation of Sertoli cell function by germ cell-secreted proteins, a major field of research in the 1980s and 1990s, was considered to have reached a dead end [for a review see [3]].

Secreted factors constitute a major class of proteins defining the cellular secretome and acting as regulators of numerous biological and physiological processes, through paracrine or autocrine effects. Secretomics has, thus, rapidly become a key area of proteomics research, for the discovery of biomarkers and therapeutic targets in diseases [50], [51]. Studies of diffusible factors are limited by: *i*) the difficulties isolating and growing many cell types in single primary cultures; *ii*) the need for coculture or cultures on specific substrates more closely reflecting the *in vivo* situation but calling for challenging experimental designs *iii*) the need to add fetal calf serum to culture medium, its removal by starvation being incomplete, masking the presence of low-abundance secreted proteins; *iv*) the use of immortalized cell lines, the secretion patterns of which may differ considerably from that of normal cells in primary culture [for a review see [52]]. Moreover, even under optimal culture conditions, it remains difficult to control cell damage and *in vitro* secretome studies have consistently shown that several intracellular proteins are released by cultured mammalian cells into the conditioned medium [53]. Many of these proteins may originate from cell death or leakage, but others may be secreted via non classical pathways, perhaps via vesicles and exosomes [54], and may have extracellular functions (e.g., α-enolase). The analysis of secretomes remains highly challenging, due to these technical issues. The development of alternative approaches is therefore required.

In this study, we developed an “integrative omics” strategy for studying the germ cell secretome through the analysis of TF, a complex biological fluid that surrounds germ and somatic cells within the seminiferous tubules and contains many secreted peptides, proteins and hormones [24], [25]. Bortoluzzi *et al*. studied the skeletal muscle secretome, using a pioneering computational approach in which putatively secreted proteins were identified by sequential sieving, signal peptide prediction, the recognition of transmembrane regions and the analysis of protein annotation [55]. Similarly, a publicly available secreted protein database, the SPD, was specifically designed to define as exhaustive as possible a secretome for humans, mice and rats, and to rank the resulting known and predicted secreted factors into different categories, according to a homemade classification pipeline [27].

We were able to link 1,056 of the 2,651 ram TF proteins and 450 of the rat TF proteins to mouse Entrez gene IDs, reflecting the stringency of our approach. The smaller number of Entrez gene IDs than of proteins identified can be accounted for by: (*i*) the inherent redundancy of the multi-species protein sequence database (subset of the UniProt Knowledgebase for mammals), leading to the identification of several orthologous proteins/genes that were eventually linked to a single mouse Entrez gene ID; (*ii*), the absence of links to the Entrez gene or HomoloGene databases for some of the proteins identified.

As expected, our dataset included several proteins known to be secreted by Sertoli cells, such as inhibin-α, and the inhibin-βA and inhibin-β subunits, with a SPD rank of 0 corresponding to known secreted proteins. As target cells for follicle-stimulating hormone (FSH) within the testis, Sertoli cells finely tune the secretion of these subunits and production of the glycoprotein hormone — consisting of two partially homologous subunits (α and β) — which downregulates FSH secretion by the pituitary gland [56]. Inhibin is thus probably produced at a low abundance, explaining the detection of inhibin subunits in ram TF but not in rat TF, for which the amounts of material available for analysis were too small. Another protein preferentially secreted by Sertoli cells was the androgen-binding protein SHBG, historically considered to be a marker of Sertoli cell function [57]. ABP, a high-affinity carrier of androgens within the seminiferous tubules, is expressed at higher basal levels and was thus detected in the TF of both rats and rams, with a SPD rank of 1, corresponding to proteins with signal peptides predicted by both the PSORT and Sec-HMMER algorithms. Other markers of Sertoli cell function detected with high secretion scores, were clusterin, α2-macroglobulin and AMH [for a review, see [3]]. Sertoli cells are easy to maintain in primary culture, and their secretome can therefore be studied by the conventional *in vitro* approach. Our strategy identified a large number of putative secreted proteins from both the germ cell and Sertoli cell lineages, but we exploited only part of this valuable dataset for this study, focusing on the germ cell secretome. We identified 66 germ cell-secreted proteins, constituting the first theoretical secretome established for testicular seminiferous tubule cells and, in particular, for germ cells. We are now working to validate these candidates and their potential partners and to understand their role in spermatogenesis.

Two of the proposed protein-protein interactions highlighted in this study were validated in PLA (Duolink). In our view, the colocalization of two proteins with specific antibodies, even by confocal microscopy, does not provide conclusive proof that the two proteins interact physically. Several approaches for assessing protein-protein interactions are available, including the well known yeast two-hybrid assay, co-immunoprecipitation and tandem affinity purification followed by mass spectrometry characterization. These techniques can yield valuable information, but they do not explain how proteins interact within cells. Förster resonance energy transfer (FRET)-based methods [58] and protein-fragment complementation assays (PCA) provide interesting alternative approaches to studying protein-protein interactions in living cells [59]. However, both these methods are based on protein tagging and the need for the time-consuming production of molecular constructs (for a review see [60]). For this reason, we used the protein ligation assay, which has a number of advantages over these methods because it can be used for *in situ* interaction analyses for any protein for which specific antibodies are available. In this context, recognition by two or more binding reagents ensures a high degree of specificity [60].

The interaction between APOH and CDC42 has been demonstrated before, with yeast two-hybrid technology [44]. These two proteins play crucial roles in spermatogenesis, but their interaction in the testis has never before been reported. CDC42 is a plasma membrane-associated small GTPase involved in the regulation of Sertoli cell polarity, cell adhesion at both the blood-testis barrier and the apical ectoplasmic specialization structure, and regulation of the dynamics of the blood-testis barrier [61], [62]. Beta-2-glycoprotein 1, officially known as APOH, is a protein that binds to various kinds of negatively charged substances, such as phospholipids, thus playing a critical role in the clearance of liposomes from the blood [63], [64]. It has been detected in the testis, at a time point corresponding to the appearance of mature germ cells and the completion of spermatogenesis, suggesting a role in apoptotic body clearance during spermatogenesis [65], [66]. We found that APOH was detectable in TF, secreted by meiotic/post-meiotic germ cells and potentially interacted with CDC42 at the Sertoli cell membrane. Cell junctions in the seminiferous epithelium are dynamic structures involved in signal transduction events [67], so the APOH/CDC42 interaction may be involved in communication between germ cells and Sertoli cells at late stages of spermatogenesis, thereby facilitating the endocytosis of residual bodies by Sertoli cells.

The NGFR, also known as p75NTR, is an alternative receptor to APP in neurons [43], [68]. The testis has been shown to contain both these proteins, but their interaction has never before been reported in this organ. APP is a transmembrane protein, the cleavage of which generates peptides, some of which are associated with Alzheimer's disease. APP is produced by cells with a high membrane fusion activity (*i.e*., membrane turnover activity), including Sertoli cells [69], suggesting a role in the maintenance of cellular integrity. In other studies, APP has been detected in the acrosome and the growing tail of spermatids in rat seminiferous tubules [70] and in the head and tail of human spermatozoa [71], suggesting a role in sperm function. Several studies have reported the expression of NGFR in various testicular cell types, including pachytene spermatocytes and round spermatids [72], [73]. We show here that APP, which is potentially secreted into the TF by Sertoli cells, interacts with NGFR at the membrane of meiotic and post-meiotic germ cells, consistent with a potential role in the regulation of male germ cell development and spermiogenesis.

Our results provide new insight into the extracellular factors potentially involved in correctly establishing essential communication between germ cells and Sertoli cells. Using a conventional *in vitro* culture approach, Flenkenthaler *et al*. [74] recently established the secretome of human testicular peritubular cells and suggested that these cells played a crucial role in maintaining an appropriate microenvironment in the spermatogonial stem cell niche, through the secretion of proteins involved in cell adhesion and migration. We show here that combining three types of omics data results in the accurate identification of potential cell-specific diffusible factors in a complex biological fluid composed of secretions from several cell types. One has to keep in mind that spermatogenesis is a highly synchronized process in which fine regulation takes place along the cycle of the seminiferous epithelium. As a consequence, cell secretomes should vary at each stage of the spermatogenic cycle, leading to a dynamic and specific protein composition of the TF microenvironment along the tubules. In such context, only a global secretome analysis of seminiferous tubules cells could be performed here. Indeed, the approach used was not meant to characterize proteins involved in Sertoli-germ cells communication at each specific stage of the spermatogenic cycle. Moreover the protein dataset presented here is that of the TF collected at the rete testis. It could be slightly different from that of a TF collected elsewhere, as the rete testis is atypical of seminiferous tubules, as specific functions and is likely to contain specific proteins. Our results confirm nevertheless that it is now possible to study cell secretomes that were previously inaccessible, by a computational approach overcoming the limitations of *in vitro* culture-based methods. We are currently investigating these interactions further, by conventional biological and biochemical approaches, and are also validating several other potential partners, to increase our understanding of the crosstalk between germ cells and Sertoli cells during spermatogenesis. This integrative strategy could be used more widely, to study other cell secretomes and to elucidate meaningful molecular mechanisms underlying cell-cell communication.

## Materials and Methods

### Animals and collection of testicular rete testis fluids

Animal experimentations were approved by the local Veterinary Departments of the Departmental Direction of the Protection of the Populations (Ille et Vilaine DDCSPP, Rennes, France and Indre et Loire DDPP, Tours, France). Sprague-Dawley rats for the various experiments were purchased from Elevage Janvier (Le Genest Saint Isle, France). Ile-de-France rams were provided by the INRA-UMR85 experimental unit (Nouzilly, France). Rat TF was collected by microsurgery 24 h after rete testis ligature, as previously described [75]. Ram TF was collected from several animals by *in vivo* rete testis cannulation, as previously described [36]. TF was collected from each ram over a period of 12 hours. Spermatozoa were separated by centrifugation at 15000×*g* for 20 minutes at 4°C. The supernatant was stored at −20°C until use. Proteomic analysis was carried out with a pool of equal amounts of TF from five rams.

### Protein separation and digestion

TF was thawed and centrifuged for 15 minutes at 3000×*g* and protein concentration was determined by the Bradford method (Bio-Rad, Marnes-la-Coquette, France). Samples were fractionated by SDS-PAGE in a 12% acrylamide precast gel (Invitrogen, Saint Aubin, France). The gel was then stained with Coomassie Blue G-250, with the EZBlue gel-staining reagent (Sigma-Aldrich, Saint-Quentin Fallavier, France). The entire gel lane was excised and cut into 20 bands, which were washed with various ACN/100 mM NH_4_HCO_3_ solutions. In-gel digestion was performed overnight at 37°C with modified trypsin (Promega, Charbonnières Les Bains, France), according to a previously described protocol [76]. Proteolytic peptides were then extracted from the gel by sequential incubation, using routine procedures, and extracts were concentrated to a final volume of 20 µl by evaporation.

### Mass spectrometry analysis

Peptide mixtures were analyzed with a nanoflow high-performance liquid chromatography (HPLC) system (LC Packings Ultimate 3000, Thermo Fisher Scientific, Courtaboeuf, France) connected to a hybrid LTQ-OrbiTrap XL (Thermo Fisher Scientific) equipped with a nanoelectrospray ion source (New Objective), as previously described [77]. The mass spectrometer was operated in the data-dependent mode by automatic switching between full-survey scan MS and consecutive MS/MS acquisition. Survey full-scan MS spectra (mass range: 400–2000) were acquired in the OrbiTrap section of the instrument, with a resolution of *r* = 60,000 at m/z 400; ion injection times were calculated for each spectrum, to allow for the accumulation of 10^6^ ions in the OrbiTrap. The seven peptide ions giving the most intense signal in each survey scan, with an intensity above 2000 counts (to avoid triggering fragmentation too early in the peptide elution profile) and a charge state ≥2 were sequentially isolated at a target value of 10,000 and fragmented in the linear ion trap by collision-induced dissociation. The normalized collision energy was set to 35%, with an activation time of 30 milliseconds. Peaks selected for fragmentation were automatically placed on a dynamic exclusion list for 120 s with a mass tolerance of ±10 ppm, to prevent the selection of the same ion for fragmentation more than once. The following parameters were used: the repeat count was set to 1, the exclusion list size limit was 500, singly charged precursors were rejected, and a maximum injection time of 500 ms and 300 ms for full MS and MS/MS scan events, respectively, was set. For an optimal duty cycle, the fragment ion spectra were recorded in the LTQ mass spectrometer in parallel with OrbiTrap full-scan detection. For OrbiTrap measurements, an external calibration was used before each injection series, giving an overall error for mass accuracy of less than 5 ppm for the detected peptides. MS data were saved in RAW file format (Thermo Fisher Scientific), with XCalibur 2.0.7 version 2.4.

### Protein identification

Data were analyzed with Proteome Discoverer 1.2 software, with the Mascot (Matrixscience) and SEQUEST database search engines for peptide and protein identification. MS/MS spectra were used as queries to search the UniProt Database (Release 2010_07) restricted to Mammalia (121,346,285 residues; 330267 sequences). Mass tolerance was set to 10 ppm and 0.5 Daltons for MS and MS/MS, respectively. Enzyme selectivity was set to full trypsin, with one missed cleavage allowed. The allowed protein modifications were fixed carbamidomethylation of cysteines and variable oxidation of methionine, and variable phosphorylation of serine, threonine and tyrosine. Identified peptides were filtered on the basis of Xcorr values and Mascot score, to obtain a false discovery rate of 1% and a false positive rate of 5%. Proteome Discoverer was used to create a unique list of identified proteins per band. Redundancy was avoided by grouping proteins with shared peptides and displaying only the protein with the best score or the highest sequence coverage for a given group.

### Data repository

Mascot protein identification data (.dat files) were converted into PRIDE xml files with the Pride Converter 2 Tool Suite (v.2.0.19,[78]) and submitted to the PRIDE Proteomics Identifications database (http://www.ebi.ac.uk/pride; [79]). Data are accessible under the project names “Proteomic characterization of ram testicular fluid” and “Proteomic characterization of rat testicular fluid” (accession number: 31052-31111).

### Proteomic data analysis

For definition of the mammalian TF proteome (Fig. 2, panel A), all UniProt accession numbers corresponding to the proteins identified were linked to their corresponding gene IDs (NCBI Entrez gene IDs), which were subsequently mapped to the corresponding mouse gene IDs by HomoloGene [80] and to OMA [81] IDs with Annotation, Mapping, Expression and Network (AMEN) analysis software [38]. *Mus musculus* was selected as the reference species because more extensive annotation data are available for this species than for rat or sheep.

### Transcriptomic data analysis

For definition of the transcriptomes of both Sertoli and germ cells (Fig. 2, panel B), we made use of a transcriptomic dataset including two populations of germ cells enriched in different types of cell (pachytene spermatocytes and round spermatids) and one population of Sertoli cells, based on a previously published Affymetrix GeneChip microarray analysis (Mouse 430 2.0 and rat 230 2.0 GeneChips) on mouse and rat [26] (ArrayExpress ID: E-TABM-130). Data were analyzed with AMEN. For identification of the testicular transcripts preferentially expressed in germ cells rather than Sertoli cells, we selected probe sets if at least one intensity value across germline samples (spermatocytes or spermatids) exceeded the background expression cutoff (BEC∼6.1, corresponding to the overall median log2-transformed intensity). We avoided the inclusion of probe sets with signal values close to the BEC, by selecting only those with intensities at least twice the BEC. A LIMMA statistical test (F-value adjusted by the false discovery rate method: *p*≤0.01) was used to select probe sets displaying statistically significant changes. A similar strategy was used to define the Sertoli cell transcriptome, with the selection of probe sets yielding a signal above the BEC in Sertoli cell samples, with a fold-change ≥2.0 with respect to the germ cell signal and a *p*-value≤0.01. The selected probe set IDs were then linked to the corresponding gene IDs (NCBI Entrez gene IDs) with AMEN. Rat gene IDs were sequentially converted into OMA and HomoloGene IDs and then, finally, into mouse gene IDs. Any mouse gene IDs associated with several probe set IDs with opposite expression patterns (e.g. in both the germ cell and Sertoli cell transcriptomes) were discarded from the analysis.

### Secretome and membranome data analysis

For the definition of a set of proteins actively secreted outside the cell membrane, otherwise known as a secretome (Fig. 2, panel C), we made use of a comprehensive collection of mouse secreted proteins via a web-accessible resource called SPD [27]. This database consists of a core dataset of ∼18,000 secreted proteins retrieved from Swiss-Prot/TrEMBL, Ensembl, RefSeq, and ranked according to the confidence associated with the prediction of their secretion, from rank 0 to 3. Briefly, proteins of rank 0 correspond to known secreted proteins in Swiss-Prot; rank 1 corresponds to proteins with a signal peptides predicted by both PSORT and Sec-HMMER; rank 2 corresponds to proteins with signal peptides predicted by either PSORT or Sec-HMMER; and rank 3 corresponds to proteins with a signal peptide of more than 70 amino acids predicted by Sec-HMMER only. We did not consider rank 3 proteins in our analysis. Mouse protein IDs of ranks 0-2 were converted into their corresponding mouse Entrez gene IDs.

Human, mouse and rat genes encoding membrane proteins, corresponding to the membranome, were selected on the basis of their association with the “cell surface” (GO:0009986) and “plasma membrane” (GO:0005886) Gene Ontology terms in the ‘gene2go’ file downloaded from the NCBI website (Fig. 2, panel D). The selected human and rat gene IDs were converted to the corresponding mouse gene IDs via HomoloGene and OMA IDs.

### Integrative omics data analysis

The germ cell (or Sertoli cell) secretome (Fig. 2, panel E) — the genes (mouse Entrez gene IDs) expressed in germ cells (or Sertoli cells) and encoding proteins actively secreted into TF — was determined by the intersection of three types of omics data: (i) the germ cell (or Sertoli cell) transcriptome; (ii) the secretome; and (iii) the TF proteome.

The Sertoli cell (or germ cell) membranome (Fig. 2, panel E) — the genes (mouse Entrez gene IDs) expressed in Sertoli cells (or germ cells) and encoding membrane proteins — was determined by the intersection of two types of omics data: (i) the Sertoli cell (or germ cell) transcriptome; and, (ii) the membranome.

Finally, we investigated dialog between germ cells and Sertoli cells by focusing on interactomic data describing physical interactions between the proteins of the secretome of one type of cell (germ cell or Sertoli cell) and those of the membranome or secretome of the other type of cell.

### Interactomic data analysis

Physical protein-protein interaction data were downloaded from the BioGRID, HPRD, IntAct, MINT and NCBI databases [28]–[31]. The physical associations explored in this study correspond to a consolidation of all mammalian datasets. Briefly, all mammalian protein IDs were converted into mouse gene IDs via the OMA and HomoloGene databases. A representation of the network was drawn with AMEN software and edited by hand.

### *In situ* proximity ligation assay (PLA)

Bouin reagent-fixed and paraffin-embedded rat testes were prepared as previously described [82]. Antigens were retrieved by heating for 10 minutes in a microwave oven, in citrate buffer (10 mM, pH 6.0, 0.05% Tween 20) for APOH/CDC42 couple, or in Tris-EDTA buffer (10 mM Tris, 1 mM EDTA, pH 9.0, 0.05% Tween 20) for APP/NGFR. Antibodies directed against ApoH (LS-B2591 goat polyclonal antibody, LifeSpan Biosciences, Inc., Seattle, USA), CDC42 (ab64533 rabbit polyclonal antibody, Abcam, Cambridge, UK), APP (rabbit polyclonal antibody, Abnova, Taipei City, Taiwan) and NGFR (mouse monoclonal antibody, Osenses Pty Ltd, Keswick, Australia) were used at the following concentrations: 2.5 µg/ml, 5 µg/ml, a dilution of 1/200 and 5 µg/ml respectively. We used a Duolink II *in situ* PLA kit with PLA probes anti-rabbit PLUS and anti-goat MINUS or PLA probes anti-rabbit PLUS and anti-mouse MINUS (OLINK Bioscience, Uppsala, Sweden) for the detection of APOH/CDC42 or APP/NGFR interactions *in situ*, respectively, according to the manufacturer's instructions. Complex formation was detected with Duolink II Detection Reagents Far Red (OLINK Bioscience, Uppsala, Sweden) and a DMRXA2 microscope (Leica Microsystèmes SAS, Nanterre, France).

## Supporting Information

Table S1 **Lists the core reference proteome of the mammalian testicular fluid (TF).**
(XLSX)
